# Donor-Derived Cell-Free DNA for Kidney Allograft Surveillance after Conversion to Belatacept: Prospective Pilot Study

**DOI:** 10.3390/jcm12062437

**Published:** 2023-03-22

**Authors:** Bilgin Osmanodja, Aylin Akifova, Michael Oellerich, Julia Beck, Kirsten Bornemann-Kolatzki, Ekkehard Schütz, Klemens Budde

**Affiliations:** 1 Department of Nephrology and Intensive Care, Charité—Universitätsmedizin Berlin, Charitéplatz 1, 10117 Berlin, Germany; 2 Department of Clinical Pharmacology, University Medical Center Göttingen, 37075 Göttingen, Germany; 3 Chronix Biomedical GmbH, 37073 Göttingen, Germany

**Keywords:** donor-derived cell-free DNA, kidney transplantation, biomarkers, graft rejection, immunosuppressive agents, acute kidney injury, belatacept

## Abstract

Donor-derived cell-free DNA (dd-cfDNA) is used as a biomarker for detection of antibody-mediated rejection (ABMR) and other forms of graft injury. Another potential indication is guidance of immunosuppressive therapy when no therapeutic drug monitoring is available. In such situations, detection of patients with overt or subclinical graft injury is important to personalize immunosuppression. We prospectively measured dd-cfDNA in 22 kidney transplant recipients (KTR) over a period of 6 months after conversion to belatacept for clinical indication and assessed routine clinical parameters. Patient and graft survival was 100% after 6 months, and eGFR remained stable (28.7 vs. 31.1 mL/min/1.73 m^2^, *p* = 0.60). Out of 22 patients, 2 (9%) developed biopsy-proven rejection—one episode of low-grade TCMR IA and one episode of caABMR. While both episodes were detected by increase in creatinine, the caABMR episode led to increase in absolute dd-cfDNA (168 copies/mL) above the cut-off of 50 copies/mL, while the TCMR episode did show slightly increased relative dd-cfDNA (0.85%) despite normal absolute dd-cfDNA (22 copies/mL). Dd-cfDNA did not differ before and after conversion in a subgroup of 12 KTR with previous calcineurin inhibitor therapy and no rejection (12.5 vs. 25.3 copies/mL, *p* = 0.34). In this subgroup, 3/12 (25%) patients showed increase of absolute dd-cfDNA above the prespecified cut-off (50 copies/mL) despite improving eGFR. Increase in dd-cfDNA after conversion to belatacept is common and could point towards subclinical allograft injury. To detect subclinical TCMR changes without vascular lesions, additional biomarkers or urinary dd-cfDNA should complement plasma dd-cfDNA. Resolving CNI toxicity is unlikely to be detected by decreased dd-cfDNA levels. In summary, the sole determination of dd-cfDNA has limited utility in the guidance of patients after late conversion to belatacept. Further studies should focus on patients undergoing early conversion and include protocol biopsies at least for patients with increased dd-cfDNA.

## 1. Introduction

Donor-derived cell-free DNA (dd-cfDNA) is an emerging biomarker in kidney transplantation. It is currently used as a diagnostic test for antibody-mediated rejection (ABMR) and T-cell-mediated rejection (TCMR) [1,2,3,4,5]. Other suggested indications are guidance of immunosuppressive therapy, when no therapeutic drug monitoring (TDM) is available, for tapering of calcineurin inhibitors (CNI) or in clinically challenging dilemmas such as BK-nephropathy [6,7]. In those situations, detection of patients with overt or subclinical graft injury may support personalized immunosuppression.

CNI have substantially improved graft survival but have several adverse effects, including nephrotoxicity, neurotoxicity, as well as cardiovascular side effects such as hypertension, dyslipidemia, and increased risk for post-transplant diabetes mellitus (PTDM) [8,9,10,11,12]. To avoid CNI, belatacept has been developed as an alternative immunosuppressant. Belatacept is a fusion protein of human IgG1 Fc-fragment and CTLA-4, mimicking the latter’s inhibitory effects on T-cell co-stimulation. It has been studied as primary immunosuppressant and as a CNI alternative in the later post-transplant phase [13,14]. When administered immediately after transplantation, a higher rate of TCMR of 17–22% in comparison to 7% for cyclosporine and a higher risk for post-transplant lymphoproliferative disorders (PTLD) involving the central nervous system have been observed in the first year [13]. When converting from CNI to belatacept more than 6 months after transplantation, 8% of kidney transplant recipients (KTR) developed TCMR in the belatacept group and 4% in the CNI group during the first year after conversion. In both settings, belatacept led to overall improvement of estimated glomerular filtration rate (eGFR) [14].

With uniform dosing and no TDM being available for KTR treated with belatacept, there is currently no way to detect over- or underimmunosuppression and prevent the respective consequences of rejection or infection. Since dd-cfDNA is able to detect allograft injury, it was hypothesized that dd-cfDNA could help to determine the minimal necessary immunosuppression [6]. In line with this rationale, two studies are currently investigating whether a combination of dd-cfDNA and whole-blood transcriptome analysis are able to detect patients who are suitable for belatacept monotherapy (NCT04177095, NCT04786067).

The aim of the present study was to assess dd-cfDNA and clinical outcomes in KTR who underwent conversion to belatacept for clinical indication. Our main hypothesis was that for patients with biopsy-proven or suspected CNI toxicity, dd-cfDNA decreases after discontinuing the CNI due to resolving toxicity. Furthermore, we wanted to explore the proportion of patients with increased dd-cfDNA after conversion to standard-dose belatacept and the corresponding clinical outcomes including eGFR changes and biopsy-proven rejection episodes.

## 2. Methods

We enrolled 22 KTR who underwent conversion of immunosuppressive medication to belatacept for clinical indication from April 2020 until July 2022. At baseline, we collected donor data (age, sex, and living versus deceased donation), recipient data (age, sex, cause of chronic kidney disease, type of dialysis, duration of dialysis, induction immunosuppressive regimen, and time since transplantation), and clinical data (latest biopsy results and immunosuppressive regimen) from our proprietary electronic health record and transplant database *TBase* [15]. The patients received regular follow-up visits with laboratory assessments including plasma creatinine, estimated glomerular filtration rate (eGFR), and albumin-creatinine-ratio (ACR) as standard of care (SOC) at baseline and after one, three, and six months. Additionally, de novo donor-specific antibody (dnDSA) formation was assessed once per year in all patients, as previously described [16,17,18]. We assessed main clinical events (acute kidney injury, biopsy-proven rejection episodes, and death) as well.

In addition to SOC, the patients received dd-cfDNA testing (Chronix Biomedical, Göttingen, Germany) at baseline and after one, three, and six months. The 6-month observation period was chosen since most rejections occurred during this timeframe in previous trials [14].

Tapering of previous immunosuppression was performed as summarized in Table S1. In the meantime, belatacept was initiated according to Rostaing et al. [19] Belatacept 5 mg/kg was given by intravenous infusion on days 1, 15, 29, 43, and 57 and then every 28 days thereafter.

Measurement of dd-cfDNA was performed as described previously [1,20]. In brief, for each patient, four informative single-nucleotide polymorphisms (SNPs), defined as a SNP for which the recipient has a homozygous allelic state, and the graft carries at least one heterozygous allele, were selected from a predefined set of 40 SNPs. These four SNPs were used to quantify the dd-cfDNA (%) concentration, which is defined as donor-alleles/(donor-alleles + recipient-alleles). Results for SNPs with heterozygous graft genotypes were corrected by a factor of two. Total cfDNA was extracted from up to 8 mL plasma collected in certified blood collection tubes (Streck Corp., Omaha, NE, USA). The concentration was determined using droplet-digital PCR (ddPCR) and was corrected for extraction loss and cfDNA fragmentation as described previously [1]. Absolute concentration of dd-cfDNA per mL plasma was calculated by multiplying total cfDNA (copies/mL) and dd-cfDNA (%). An abnormal dd-cfDNA result was defined as a value of >50 copies/mL for absolute and >0.5% for relative quantification [1,21].

The institutional review board of the ethics committee of Charité-Universitätsmedizin Berlin, Germany, approved the study (approval number EA2/144/20), and all procedures were in accordance with the 1964 Helsinki Declaration and its later amendments or comparable ethical standards. Written informed consent was obtained from all patients. Statistical analysis was performed using R version 4.1.2.

## 3. Results

In total, 22 patients were enrolled from April 2020 until July 2022. As maintenance immunosuppression, 17/22 (77%) received tacrolimus, 4/22 (18%) received cyclosporine, and 1 patient (5%) received sirolimus before conversion to belatacept. Overall, 20 of 22 patients (91%) were converted in the later post-transplant period (>1 year after transplantation), and 15 of 22 patients (68%) had severe arteriolar hyalinosis (ah3) as a sign of chronic CNI-toxicity in the latest biopsy. Additionally, 1/22 patients (5%) showed moderate arteriolar hyalinosis (ah2), and 3/22 patients (14%) showed acute tubular necrosis (ATN) attributed to acute CNI toxicity in the latest biopsy. Four patients (18%) underwent empiric conversion to belatacept due to suspected CNI-toxicity without performing kidney biopsy: three patients refused biopsy due to long transplant age of 18, 20, and 23 years, respectively, and for another patient, biopsy could not be obtained due to dual antiplatelet therapy. In 8/22 patients (36%), ABMR was proven or suspected in the latest kidney allograft biopsy. Patient characteristics are summarized in Table 1 and detailed in Table S2.

**Figure 1:**
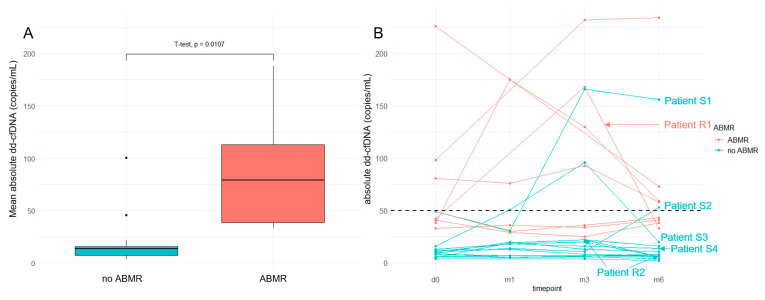
(**A**) Patients with previous diagnosis of ABMR show higher mean absolute dd-cfDNA over the study period of 6 months. (**B**) Absolute dd-cfDNA in patients undergoing conversion to belatacept. Six patients are highlighted. R, clinical rejection episode; S, clinically stable/improving; ABMR, antibody-mediated rejection.

After conversion to belatacept, eGFR remained stable in our cohort from baseline until month 6 (28.7 vs. 31.1 mL/min/1.73 m^2^, *p* = 0.60). All dd-cfDNA and total cfDNA measurements as well as creatinine and ACR values are provided in Table S1. Missing values occurred in 7 out of 88 scheduled measurements (8%). Importantly, patients with ABMR had higher mean absolute dd-cfDNA over the study period than patients without ABMR (88 vs. 20 copies/mL, *p* = 0.01) (Figure 1A). A total of 3/8 patients with ABMR had absolute dd-cfDNA values above the prespecified cut-off of >50 copies/mL before conversion, while the other 5 patients had values in the upper normal range. During the course of the study, all patients with ABMR always had values ≥ 25 copies/mL, and 5/8 patients with ABMR were at least temporarily above the cut-off (Figure 1B).

**Figure 2:**
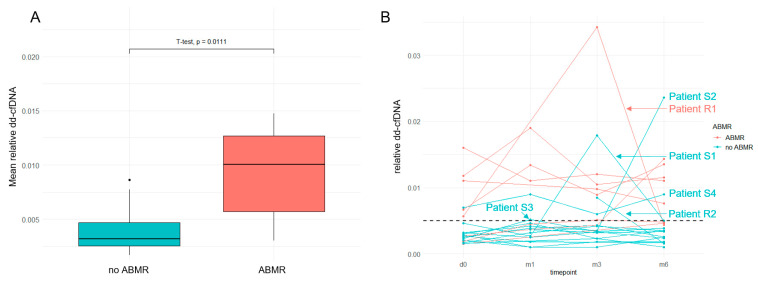
(**A**) Patients with previous diagnosis of ABMR show higher mean relative dd-cfDNA over the study period of 6 months. (**B**) Relative dd-cfDNA in patients undergoing conversion to belatacept. Six patients are highlighted. R, clinical rejection episode; S, clinically stable/improving; ABMR, antibody-mediated rejection.

Comparable results were found when using relative dd-cfDNA and the prespecified cut-off of 0.5%. The mean relative dd-cfDNA over the study period was higher in patients with previous ABMR than in patients without ABMR (0.92% vs. 0.40%, *p* = 0.01) (Figure 2A). A total of 7/8 patients with previous ABMR had at least one relative dd-cfDNA measurement above the cut-off over the study period (Figure 2B). 

During the observation period, two episodes of biopsy-proven rejection occurred in 22 patients (9%). One episode of caABMR (Patient R1 in Figure 1B and Figure 2B) was accompanied by increase in creatinine (2.5 to 3.7 mg/dL) and dd-cfDNA (42 to 168 copies/mL), and one episode of TCMR IA (Patient R2 in Figure 2B) was indicated by increase in creatinine (1.73 mg/dL to 2.78 mg/dL), absolute dd-cfDNA levels below the cut-off (22 copies/mL at the time of biopsy), and relative dd-cfDNA levels above the cut-off (0.85% at the time of biopsy). While dnDSA were existent in 9/22 patients (41%) before switch to belatacept, no additional dnDSA formation or changes in specificity were observed after switch to belatacept in any of the 22 patients.

From twelve patients with previous CNI therapy and without rejection (subgroup A), three patients (25%) developed increase in absolute dd-cfDNA above the cut-off (Patients S1–S3 in Figure 1B and Figure 2B). Another patient (Patient S4 in Figure 2B) showed increased relative dd-cfDNA but not absolute dd-cfDNA over the entire study period. None of these four patients underwent indication biopsy—Patient S1 was under dual antiplatelet therapy and showed improving creatinine (4.72 to 3.62 mg/dL), Patient S2 showed slightly increased dd-cfDNA (53 copies/mL) with improving kidney function (creatinine 1.88 to 1.33 mg/dL), Patient S3 showed only transient dd-cfDNA increase (96 copies/mL at month 3, 20 copies/mL at month 6) and improving creatinine (3.59 to 3.1 mg/dL), and Patient S4 showed normal absolute dd-cfDNA and improving creatinine (2.46 to 2.15 mg/dL). Hence, the cause for dd-cfDNA increase remains undetermined in these patients.

To assess whether CNI cessation leads to decreased levels of dd-cfDNA, we included only patients with CNI therapy before conversion to belatacept and excluded all patients with rejection from the subsequent analysis (subgroup A). In the remaining 12 patients, absolute dd-cfDNA levels before and 6 months after conversion to belatacept did not differ significantly (mean 12.5 vs. 25.3 copies/mL, *p* = 0.34). This was also the case when further restricting the analysis to the seven patients with improved eGFR (subgroup B) defined as higher eGFR at month 6 than before conversion (mean 15.4 vs. 38.7 copies/mL, *p* = 0.31). For relative dd-cfDNA, difference was found after CNI cessation neither in subgroup A (0.31% vs. 0.52%, *p* = 0.28) nor subgroup B (0.35% vs. 0.72%, *p* = 0.25). Moreover, no difference in total cfDNA was found in subgroup A (3893 vs. 6101 copies/mL, *p* = 0.40) or in subgroup B (4477 vs. 7231 copies/mL, *p* = 0.54).

## 4. Discussion

In this pilot study, we report the first use of dd-cfDNA for graft surveillance in KTR after conversion to belatacept-based immunosuppression. Our initial hypothesis was that we could detect resolving CNI toxicity by decreasing levels of dd-cfDNA. However, in this small cohort of patients who mostly underwent late conversion to belatacept due to chronic CNI toxicity, we detected no difference in absolute or relative dd-cfDNA levels before and after conversion in non-rejecting patients. This suggests that plasma dd-cfDNA is not suited to detect subtle changes in graft injury due to CNI toxicity, which has probably two main reasons: chronic CNI toxicity with hyalinosis of the arteriolar walls is a slowly developing process, and acute CNI toxicity mostly affects the tubular cells. Due to the short half-life of cell-free DNA in general and the mainly endothelial origin of plasma dd-cfDNA, both forms are unlikely to be accompanied by a significant increase in dd-cfDNA. Additionally, immune activation during the conversion phase can further alter dd-cfDNA levels, making it even harder to detect subtle changes. Previously, Schütz et al. showed that total cfDNA decreases over time after transplantation, which leads to an apparent increase in relative dd-cfDNA despite stable absolute dd-cfDNA [22]. Such increase was also observed in the Trifecta study, where older grafts showed higher relative dd-cfDNA [5]. It was hypothesized that this effect is due to reduced CNI exposure and subsequent increase in leukocyte stability [22] because both CNI and mTOR inhibitors have a negative effect on cell stability [23,24]. In contrast, we were not able to find a decrease in total cfDNA after CNI cessation in this cohort.

In line with previous studies, mean dd-cfDNA was higher in patients with preexisting ABMR than in those without ABMR [1,2,3,4,5]. While in our study, a recurrent ABMR episode was detected by increase in creatinine and also led to increase of dd-cfDNA, other studies indicate that dd-cfDNA increases also can precede clinical rejection [25]. Due to its ability to detect vascular graft injury, dd-cfDNA is currently discussed as an activity marker in ABMR [26]. Therefore, increased dd-cfDNA could indicate underimmunosuppression and active rejection in patients with ABMR who undergo conversion to belatacept for concomitant CNI toxicity. Previously, we have demonstrated, ongoing microvascular inflammation was a risk factor for graft loss after conversion to belatacept, while the presence of dnDSA and chronic ABMR was not [27]. Furthermore, no additional dnDSA formation was observed in our study, which is in line with the reduced rate of dnDSA formation after conversion to belatacept in comparison to CNI-based regimens [13,14]. However, due to the absence of evidence-based therapy options for ABMR, the clinical consequences are uncertain and warrant further investigation [28,29].

The rate of TCMR in our study was comparable to previous studies, although it is important to note that rejection frequency depends on time after transplantation and the proportion of patients with previous TCMR and ABMR [13,14,30]. TCMR episodes without vascular lesions are not reliably detected by plasma dd-cfDNA since inflammation occurs predominantly in the tubulointerstitial compartment [26]. This was shown exemplarily in our study, where an episode of low-grade TCMR (Banff IA) led to slight increase in relative dd-cfDNA but no increase in absolute dd-cfDNA. However, in the Trifecta study, patients with TCMR-related transcriptomic changes showed increased dd-cfDNA with a median of 1.61%, while patients with histological TCMR diagnosis had median dd-cfDNA of 0.88% [5]. For low-grade TCMR episodes that occur after conversion to belatacept, the potential benefit of dd-cfDNA for graft surveillance is reduced. Urinary dd-cfDNA and other novel biomarkers such as whole-blood transcriptome analyses are potentially better suited to detect subclinical TCMR. Combining those with dd-cfDNA could potentially help to differentiate subclinical graft injury, which is currently being studied in two trials (NCT04177095, NCT04786067).

Interestingly, three clinically improving patients without previous ABMR showed increased absolute dd-cfDNA, and another patient showed increased relative dd-cfDNA, none of which was accompanied by deteriorating renal function. Consequently, no indication biopsies were performed for these patients, leaving the reasons undetermined. However, such patients are of particular interest since they could experience subclinical graft injury due to rejection (ABMR, TCMR), infectious complications (e.g., BKV), or other causes and may need a personalized immunosuppressive regimen. While some of these changes were subtle, further studies may determine the clinical relevance by scheduling protocol biopsies in patients with increased dd-cfDNA. While for the assay used in this study, a cut-off of 50 copies/mL may be adequate to guide protocol biopsies, other assays may use different cut-offs. Sample size calculations can assume that 25% of KTR without rejection who undergo conversion from CNI to belatacept will show at least transient increases in absolute dd-cfDNA above prespecified cut-offs.

## 5. Limitations

The main limitations of this study are its small sample size and the lack of follow-up biopsies in patients with increased dd-cfDNA after conversion to belatacept. Another limitation is the limited amount of KTR undergoing early conversion to belatacept in this study. This could lead to false-negative dd-cfDNA results due to a high grade of interstitial fibrosis in older allografts. Advanced kidney lesions together with small numbers and a heterogeneous patient population explain that we did not observe a significant increase in eGFR after conversion to belatacept, contrary to most studies [13,14,30].

## 6. Conclusions

Despite its several limitations, this small pilot study indicates where to seek potential applications for dd-cfDNA for graft surveillance in the future. To detect subclinical TCMR changes in patients undergoing conversion to belatacept, additional biomarkers or urinary dd-cfDNA should complement plasma dd-cfDNA to enable detection of TCMR IA and IB. Furthermore, we suggest studying such combinations of biomarkers mostly in patients undergoing early conversion to belatacept to reduce the possibility of false-negative results due to high grades of interstitial fibrosis. Such future studies may also need to include protocol biopsies, at least in patients with increased dd-cfDNA, to further characterize the type of subclinical graft injury.

## Figures and Tables

**Table 1:** Demographics and baseline characteristic of 22 kidney transplant recipients who underwent conversion to belatacept due to clinical indication *.

Patient Count	22
**Demographics**	
Patient age in years (IQR)	53 (36–59)
Patient sex (female/male)	16 (73%) vs. 6 (27%)
**Clinical history**	
Primary disease - Glomerulonephritis - Genetic disease - Hypertensive or diabetic - Interstitial nephritis - Other - Unknown	9 (41%) 4 (18%) 2 (9%) 2 (9%) 1 (5%) 4 (18%)
Preemptive/PD/HD	3 (14%)/5 (23%)/14 (64%)
Median time on dialysis in years (IQR)	1 (1–6.5)
**Transplantation**	
Median years since transplantation (IQR) Converted in the first 6 months after transplantation (n)	9.5 (5.5–13) 2
Living vs. deceased donation AB0-incompatible	14 (64%) vs. 8 (36%) 4 (18%)
Median donor age in years (IQR)	55 (50–61)
Mean cold ischemia time in minutes +/− SD (for deceased donors)	725 +/− 284
Induction immunosuppression - Basiliximab - Rituximab + Basiliximab (for AB0-incompatible) - Unknown	15 (68%) 2 (9%) 5 (23%)
Maintenance immunosuppression - Tacrolimus/Cyclosporine/Sirolimus - MPA - Steroid	17 (77%)/4 (18%)/1 (5%) 20 (91%) 17 (77%)
Latest biopsy results before conversion (more than one can apply) - ABMR - ATN due to acute CNI toxicity - ah2/ah3 as sign of chronic CNI toxicity - No biopsy	8 (36%) 3 (14%) 1 (5%)/15 (68%) 4 (18%)

* IQR, interquartile range; SD, standard deviation; PD, peritoneal dialysis; HD, hemodialysis; MPA, mycophenolic acid; ATN, acute tubular necrosis; ah, arteriolar hyalinosis according to Banff 2017 classification; CNI, calcineurin inhibitor; ABMR, antibody-mediated rejection.

## Data Availability

All data underlying this study are included in the Supplementary data.

## Supplementary Materials

The following supporting information can be downloaded at: https://www.mdpi.com/article/10.3390/jcm12062437/s1, Table S1: Raw data underlying the analysis showing absolute donor-derived cell-free DNA (dd-cfDNA) in copies/mL, creatinine in mg/dl, albumin-creatinine ratio (ACR) in mg/g, and daily calcineurin inhibitor (CNI).; Table S2: Demographics and baseline characteristic of 22 kidney transplant recipients, who underwent switch to belatacept due to clinical indication.
