# Supplementary material for: Donor-Derived Cell-Free DNA for Kidney Allograft Surveillance after Conversion to Belatacept: Prospective Pilot Study
Source: J Clin Med. 2023 Mar 22;12(6):2437. doi: 10.3390/jcm12062437 (PMC10051604; doi:10.3390/jcm12062437)
Supplement: Supplementary file 1 [file jcm-12-02437-s001.zip › jcm-2286616-supplementary.pdf]

Table S1. Raw data underlying the analysis showing absolute donor-derived cell-free DNA (dd-cfDNA) in copies/mL, creatinine in mg/dl, albumin-creatinine ratio (ACR) in mg/g, and daily calcineurin inhibitor (CNI). ABMR – antibody-mediated rejection, IR-Tac – immediate-release tacrolimus, ER-Tac – extended-release tacrolimus, LCP-Tac – extended-release tacrolimus using MeldDose® technology, CyA – cyclosporine.

**PatientID**	**d0**	**m1**	**m3**	**m6**	**Value**	**ABMR**
1	33	36	34	41	dd-cfDNA (copies/mL)	1
1	0.67%	1.34%	0.89%	1.35%	ddcfDNA (%)	1
1	4997	2704	3887	3014	total cfDNA (copies/mL)	1
1	2.39	2.45	2.09	2.43	creatinine (mg/dl)	1
1	650	1316	1847	800	ACR (mg/g)	1
1	8 (IR-Tac)	2 (IR-Tac)	0	0	Daily CNI dose (mg)	1
2	81	76	93	58	dd-cfDNA (copies/mL)	1
2	0.27%	0.37%	0.43%	1.43%	ddcfDNA (%)	1
2	30029	20652	21611	4063	total cfDNA (copies/mL)	1
2	4.65	4.04	4.07	5.89	creatinine (mg/dl)	1
2	3668	2822	4467	2646	ACR (mg/g)	1
2	6 (IR-Tac)	1.5 (IR-Tac)	0	0	Daily CNI dose (mg)	1
3	42		168	33	dd-cfDNA (copies/mL)	1
3	1.10%		0.98%	0.76%	ddcfDNA (%)	1
3	3558		17141	4350	total cfDNA (copies/mL)	1
3	2.9	3.7	3.24	3.37	creatinine (mg/dl)	1
3	1235	1559	181	626	ACR (mg/g)	1
3	3 (IR-Tac)	0	1 (ER-Tac)	1 (ER-Tac)	Daily CNI dose (mg)	1
4	38	175	130	59	dd-cfDNA (copies/mL)	1
4	0.19%	0.45%	0.51%	0.48%	ddcfDNA (%)	1
4	19772	38583	25686	12337	total cfDNA (copies/mL)	1
4	3.8	2.8	2.65	2.38	creatinine (mg/dl)	1
4	386	1100	1112	383	ACR (mg/g)	1

4	8 (IR-Tac)	0	0	0	Daily CNI dose (mg)	1
5	5	19	22	16	dd-cfDNA (copies/mL)	0
5	0.17%	0.32%	0.42%	0.35%	ddcfDNA (%)	0
5	3246	6058	5128	4699	total cfDNA (copies/mL)	0
5	1.92	2.01	1.97	1.67	creatinine (mg/dl)	0
5	3	4	14	15	ACR (mg/g)	0
5	200 (CyA)	100 (CyA)	0	0	Daily CNI dose (mg)	0
6	12	13	8	7	dd-cfDNA (copies/mL)	0
6	0.30%	0.46%	0.23%	0.10%	ddcfDNA (%)	0
6	3927	2787	3419	11886	total cfDNA (copies/mL)	0
6	2.9	2.6	2.52	3.16	creatinine (mg/dl)	0
6	48	100	110	193	ACR (mg/g)	0
6	3 (IR-Tac)	3 (IR-Tac)	0	0	Daily CNI dose (mg)	0
7	49	30	36	43	dd-cfDNA (copies/mL)	1
7	1.18%	1.90%	1.05%	1.15%	ddcfDNA (%)	1
7	4113	1559	3594	3773	total cfDNA (copies/mL)	1
7	1.84	1.81	2.14	2.08	creatinine (mg/dl)	1
7	787	563	657	708	ACR (mg/g)	1
7	250 (CyA)	60 (CyA)	0	0	Daily CNI dose (mg)	1
8	49	31	166	156	dd-cfDNA (copies/mL)	0
8	0.46%	0.27%	1.79%	0.50%	ddcfDNA (%)	0
8	10588	11363	9278	31424	total cfDNA (copies/mL)	0
8	4.72	4.7	3.85	3.62	creatinine (mg/dl)	0
8	9	19	40	21	ACR (mg/g)	0
8	7 (LCP-Tac)	0	0	0	Daily CNI dose (mg)	0
9	10	14	11	53	dd-cfDNA (copies/mL)	0
9	0.32%	0.42%	0.35%	2.36%	ddcfDNA (%)	0
9	3024	3260	3148	2241	total cfDNA (copies/mL)	0
9	1.88	1.37	1.49	1.33	creatinine (mg/dl)	0

9	10	15	9.2	16	ACR (mg/g)	0
9	7 (ER-Tac)	2 (ER-Tac)	0	0	Daily CNI dose (mg)	0
10	6	7	7	7	dd-cfDNA (copies/mL)	0
10	0.29%	0.10%	0.10%	0.25%	ddcfDNA (%)	0
10	2207	3434	4798	2988	total cfDNA (copies/mL)	0
10	1.99	1.65	2.28	1.97	creatinine (mg/dl)	0
10	24	34	6	3	ACR (mg/g)	0
10	2 (IR-Tac)	1 (IR-Tac)	0	0	Daily CNI dose (mg)	0
11	226			73	dd-cfDNA (copies/mL)	1
11	0.15%			0.46%	ddcfDNA (%)	1
11	148259			16090	total cfDNA (copies/mL)	1
11	5.35	5.47		9.34	creatinine (mg/dl)	1
11	173	142		287	ACR (mg/g)	1
11	8 (LCP-Tac)	4 (LCP-Tac)	2 (LCP-Tac)	2 (LCP-Tac)	Daily CNI dose (mg)	1
12	4		4	2	dd-cfDNA (copies/mL)	0
12	0.16%		0.23%	0.35%	ddcfDNA (%)	0
12	2220		1917	587	total cfDNA (copies/mL)	0
12	1.84	1.94	1.75	1.88	creatinine (mg/dl)	0
12	87	54	125	111	ACR (mg/g)	0
12	6 (ER-Tac)	3 (ER-Tac)	0	0	Daily CNI dose (mg)	0
13	16	51	96	20	dd-cfDNA (copies/mL)	0
13	0.23%	0.52%	0.34%	0.39%	ddcfDNA (%)	0
13	7050	9882	28137	5083	total cfDNA (copies/mL)	0
13	3.59	3.02	2.67	3.1	creatinine (mg/dl)	0
13	56	125	290	219	ACR (mg/g)	0
13	1.5 (LCP-Tac)	1 (LCP-Tac)	0	0	Daily CNI dose (mg)	0
14	13	18	16	14	dd-cfDNA (copies/mL)	0
14	0.70%	0.90%	0.60%	0.90%	ddcfDNA (%)	0
14	1941	2015	2503	1535	total cfDNA (copies/mL)	0

14	2.46	2.27	2.43	2.15	creatinine (mg/dl)	0
14	14	18	17	15	ACR (mg/g)	0
14	2.5 (ER-Tac)	0	0	0	Daily CNI dose (mg)	0
15	6	5	6	4	dd-cfDNA (copies/mL)	0
15	0.27%	0.37%	0.32%	0.34%	ddcfDNA (%)	0
15	2326	1414	1968	1239	total cfDNA (copies/mL)	0
15	3.07	2.77	2.69	2.37	creatinine (mg/dl)	0
15	287	269	284	242	ACR (mg/g)	0
15	300 (CyA)	140 (CyA)	0	0	Daily CNI dose (mg)	0
16	9	20	13	11	dd-cfDNA (copies/mL)	0
16	0.25%	0.39%	0.43%	0.26%	ddcfDNA (%)	0
16	3746	5130	3151	4438	total cfDNA (copies/mL)	0
16	3.71	3.96	4.6	4.51	creatinine (mg/dl)	0
16	977	547	165	474	ACR (mg/g)	0
16	125 (CyA)	55 (CyA)	0	0	Daily CNI dose (mg)	0
17	9	5		8	dd-cfDNA (copies/mL)	0
17	0.27%	0.18%		0.17%	ddcfDNA (%)	0
17	3161	2934		4398	total cfDNA (copies/mL)	0
17	3	3.29	3.09	2.94	creatinine (mg/dl)	0
17	390	410	338	296	ACR (mg/g)	0
17	5 (ER-Tac)	1 (ER-Tac)	0	0	Daily CNI dose (mg)	0
18	11	18	20	6	dd-cfDNA (copies/mL)	0
18	0.32%	0.25%	0.33%	0.24%	ddcfDNA (%)	0
18	3275	7001	5989	2696	total cfDNA (copies/mL)	0
18	2.36	2.71	2.6	2.84	creatinine (mg/dl)	0
18	976	817	1202	870	ACR (mg/g)	0
18	2 (IR-Tac)	1 (IR-Tac)	0	1 (IR-Tac)	Daily CNI dose (mg)	0
19	41	29	25	38	dd-cfDNA (copies/mL)	1
19	1.60%	1.10%	1.20%	1.10%	ddcfDNA (%)	1

19	2562	2745	2113	3572	total cfDNA (copies/mL)	1
19	2.1	2.08	1.91	2.16	creatinine (mg/dl)	1
19	202	293	267	145	ACR (mg/g)	1
19	3 (ER-Tac)	1.5 (ER-Tac)	0	0	Daily CNI dose (mg)	1
20	8	7	7	6	dd-cfDNA (copies/mL)	0
20	0.21%	0.10%	0.18%	0.18%	ddcfDNA (%)	0
20	3742	2823	4116	3507	total cfDNA (copies/mL)	0
20	1.43	1.66	1.5	1.29	creatinine (mg/dl)	0
20	870	752	1360	898	ACR (mg/g)	0
20	0	0	0	0	Daily CNI dose (mg)	0
21			22	6	dd-cfDNA (copies/mL)	0
21			0.85%	0.16%	ddcfDNA (%)	0
21			2546	3578	total cfDNA (copies/mL)	0
21	1.73	1.84	2.78	1.78	creatinine (mg/dl)	0
21	10	22	140	34	ACR (mg/g)	0
21	3 (ER-Tac)	1 (ER-Tac)	0	0	Daily CNI dose (mg)	0
22	98		232	234	dd-cfDNA (copies/mL)	1
22	0.57%		3.42%	0.44%	ddcfDNA (%)	1
22	17073		6770	52866	total cfDNA (copies/mL)	1
22	3.13	2.78	2.3	1.95	creatinine (mg/dl)	1
22	6	8	23	46	ACR (mg/g)	1
22	18 (LCP-Tac)	4 (LCP-Tac)	0	0	Daily CNI dose (mg)	1

Table S2. Demographics and baseline characteristic of 22 kidney transplant recipients, who underwent switch to belatacept due to clinical indication.*

Patient No.	Recipient Age years /sex	Reported cause of KFRT	Dialysis modality / years on dialysis	Donor Age years/sex	Living vs Deceased Donor / AB0 compatibility	Cold ischemia time (minutes)	Induction therapy	Baseline Immuno-suppression / daily dose	Time after KTx (years)	ABMR / ah grade in latest biopsy	CAD / DM / Smoking status	Donor cause of death / comorbid conditions
1	34 / male	IgAN	PD / 2	33 / female	Living / AB0 compatible	-	Basiliximab	IR-Tac + MPA + Steroid	5	caABMR / ah3	no / no / active (5 py)	None / Hypercholesterinemia
2	32 / female	HUS	n/a	43 / female	Living / AB0 compatible	-	Basiliximab	IR-Tac + MPA + Steroid	11	caABMR / ah3	no / no / never	None / none
3	53 / male	unknown	HD / 0	50 / female	Living / AB0 compatible	-	Basiliximab	IR-Tac + MPA	7	caABMR / ah3	No / no / active (35 py)	None
4	32 / male	IgAN	HD / 1	47 / male	Living / AB0 compatible	-	Basiliximab	IR-Tac + MPA + Steroid	8	TMA from CNI toxicity or caABMR / ah3	no / no / never	None / none

5	53 / male	Alport	PD / 1	45 / male	Deceased	674	Basiliximab	CyA + MPA	13	ah2	No / yes / former (20 py)	Head trauma / none
6	69 / male	ADPKD	HD / 1	61 / female	Living / AB0 incompatible	-	Rituximab, Basiliximab	IR-Tac + MPA + Steroid	7	ATN from CNI toxicity / ah3	no / no / never	None / HTN
7	53 / male	IgAN	PD / 1	56 / female	Living / AB0 compatible	-	Basiliximab	CyA + MPA + low-dose Steroid	16	caABMR / ah3	no / no / never	None / Recurrent urinary tract infections
8	57 / male	DM	HD / 8	64 / male	Deceased	543	Basiliximab	LCP-Tac + MPA + Steroid	< 1	no biopsy – suspected CNI toxicity	yes / yes / never	Intracerebral hemorrhage / HTN, severe arteriosclerosis
9	53 / female	unknown	HD / 11	55 / female	Deceased Donor	520	Basiliximab	ER-Tac + MPA + Steroid	4	ATN from acute CNI toxicity / ah0	No / no / never	Subarachnoidal hemorrhage / HCV infection

10	62 / female	ADPKD	HD / 3	52 / male	Living / AB0 incompatible	-	Basiliximab	IR-Tac + MPA + low-dose Steroid	10	ah3	No / yes / former (50 py)	APC resistance
11	30 / female	SLE	HD / 1	52 / male	Living / AB0 incompatible	-	Rituximab, Basiliximab	LCP-Tac + MPA + Steroid	2	caABMR / ah3	no / no / never	None
12	32 / female	Sjögren's syndrome with IN	n/a	61 / female	Living / AB0 compatible	-	Basiliximab	LCP-Tac	9	ah3	no / no / never	None
13	72 / male	HTN	HD / 5	66 / male	Deceased Donor	710	Basiliximab	LCP-Tac + Steroid	2	ATN from acute CNI toxicity / ah1	Yes / yes / never	Intracerebral ischemia / HTN, multiple sclerosis, adipositas
14	76 / male	Membranous GN	HD / 1	55 / female	Living / AB0 incompatible	-	Basiliximab	ER-Tac + MPA + low-dose Steroid	10	ah3	Yes / yes / never	None
15	59 / female	IgAN	n/a	63 / male	Living	-	unknown	CyA + MPA	18	No biopsy – suspected	No / no / Former (10 py)	None

										chronic CNI toxicity		
16	68 / male	IN	HD / 5	57 / male	Deceased Donor	623	unknown	CyA + MPA	23	No biopsy – suspected chronic CNI toxicity	No / yes / never	Head trauma / None
17	57 / male	unknown	HD / 9	51 / male	Deceased Donor	1422	unknown	ER-Tac + MPA + low-dose Steroid	22	No biopsy – suspected chronic CNI toxicity	No / no / never	Intracerebral hemorrhage / unknown
18	41 / male	IgAN	PD / 1	70 / male	Living / AB0 compatible	-	unknown	IR-Tac + MPA + Steroid	10	ah3	No / no / never	Prostate cancer, HTN
19	44 / male	IgAN	PD / 1	51 / female	Living / AB0 compatible	-	Basiliximab	ER-Tac + MPA + Steroid	13	cABMR / ah3	No / no / former (3 py)	None

20	31 / male	IgAN	HD / 1	Unknown / female	Living / AB0 compatible	-	unknown	Sirolimus + MPA + Steroid	17	ah3	No / no / never	None
21	58 / male	ADPKD	HD / 9	50 / female	Deceased Donor	477	Basiliximab	ER-Tac + MPA + Steroid	9	ah3	Yes / no / never	Intracerebral hemorrhage / unknown
22	44 / male	unknown	HD / 15	61 / female	Deceased Donor	830	Basiliximab	LCP-Tac + MPA + Steroid	< 1	suspected aABMR / ah3	Yes / yes / former (15py)	Subarachnoid hemorrhage / unknown

* No. - Number, KFRT - kidney failure requiring renal replacement therapy, IgAN - IgA nephropathy, HUS - hemolytic uremic syndrome, ADPKD - autosomal dominant polycystic kidney disease, SLE – systemic lupus erythematosus, IN –interstitial nephritis, PD – peritoneal dialysis, HD – hemodialysis, KTx – kidney transplantation, IS – immunosuppression, IR-Tac – immediate release tacrolimus, ER-Tac – extended release tacrolimus, LCP-Tac – novel extended release tacrolimus, CyA – cyclosporin, MPA – mycophenolic acid, ATN – acute tubular necrosis, ah – arteriolar hyalinosis according to Banff 2017 classification, CNI – calcineurin inhibitor, caABMR – chronic active antibody mediated rejection, CAD – coronary artery disease, DM – diabetes mellitus, py – pack years, HTN – arterial hypertension
